# Mental Illness in Children: Childhood Illness and Supporting the Family

**DOI:** 10.3390/brainsci7080097

**Published:** 2017-08-08

**Authors:** Rosemary Sheehan

**Affiliations:** Department of Social Work, Monash University, Victoria 3145, Australia; rosemary.sheehan@monash.edu

## 1. Childhood Development: Life Stages and Their Impact

Childhood is a stage of life that is filled with potential for development, and the early years of childhood see immense physical changes in growth; mastery over body functions like movement; the acquisition of language and cognitive development to understand their own and others’ thinking and reasoning; and the psychosocial development of trust in the world, comfort in the care they receive from parents and caregivers, and the sense of being secure in themselves that this engenders. Bowlby placed great emphasis on the enduring emotional bonds the child needs to develop with key adults in their early life, identifying security and healthy family processes as important factors in future resilience and social competence [1]. This human development approach to child growth and development identifies what a child needs at any particular age to promote normal growth and development, to minimise risk, and foster protective factors to ensure that they thrive and prosper.

The concepts of ‘normative’ or ‘non-normative’ development are often applied to gain a sense of whether or not a child is in a ‘healthy or desirable state for someone to be in at a given age’ ([2], p. 109). When something is characterised as non-normative, it suggests that what is happening is not usual or typical, with perhaps gaps in a child’s individual functioning resulting in some subsequent impairment. When health is impaired, it can have minor to major impacts on a child’s emotional and social wellbeing. 

A child’s health status is also influenced by parental factors, such as a family’s socioeconomic situation. Poverty and low family income can adversely affect the health, education, and self-esteem of children, compromising their growth and development and general societal participation. A parent’s own ill-health, mental illness, housing instability, or social isolation can affect their capacity to effectively parent their child, provide them with supportive childhood relationships, and ensure their access to the services they need to protect them from the development of health problems. The impact of chronic stress on a child and how this can affect their development is well noted [3].

## 2. The Impact of Mental Health Problems

The prevalence of mental disorder in children in Australia finds that around one in seven (13.9%) children and adolescents aged 4–17 years, experience a mental disorder [4], equivalent to an estimated 560,000 Australian children and adolescents. Many of these children and young people are not in contact with therapeutic or support services, their mental distress left unacknowledged and without intervention, with increased risk of growing and developing with personal and social difficulties. These difficulties are frequently associated with challenges across family, education and learning, school attendance, physical health, and offending behaviour [5]. The primary health care system is typically the ‘front door’ for children with mental health concerns, with the specialist Child and Adolescent Mental Health Services offering a service for children with more severe problems. However, access to these services can be difficult, with lengthy waiting times, the need to prove eligibility for a service, and the often short-term service responses, all of which increase vulnerability and can have a devastating impact on family life [6]. 

By and large, the mental health problems children experience can be categorised as behaviour disorders, developmental disorders, and emotional disorders [6]. Behaviour (or conduct) disorders are characterised by a pattern of antisocial, aggressive, or deviant behaviour. The DSM-5 (Diagnostic and Statistical Manual of Mental Disorders) [4] states that children exhibiting these behaviours, which are more than ‘childish mischief or adolescent rebelliousness’ ([6], p. 80), are hostile or defiant in their interactions with others and may show little remorse for their behaviour. These behaviours are influenced by environment, family, and child-specific factors, such as a ‘difficult’ temperament, brain injury, chronic illness, or cognitive deficits ([6], p. 81). Attention deficit hyperactivity disorder has been a prominent explanation assigned to children who are highly distractible, hyperactive, and impulsive, and there has been controversy about its diagnostic application and reliance on drugs as an intervention. Certainly, however, there are considerable negative consequences for children who are unable to achieve at school, whose learning is significantly impaired, and whose behaviours impair their social interactions. 

Autism and Asperger’s syndrome (autism spectrum disorders) are the developmental disorders most identified in children. Autism commences before three years of age and is thought to have an organic, brain development basis. Children with autism struggle with physical contact, avoid eye contact, have speech and language disorders, do not cope with changes in their environment, and engage in repetitive behaviours. Children with Asperger’s syndrome share some features with autism in that they struggle with social interaction and fix on specific interests and behaviours, but they do not have delays in speech and cognitive development. Asperger’s syndrome is more commonly associated with boys. As adults, they can function well in employment which calls for detailed and focussed attention, but continue to have social and relational difficulties, compounded by struggling to empathise with the feelings of others. Attention deficit hyperactivity disorder (ADHD) is the most common mental disorder in Australia overall, with the Australian Australian Institute of Health and Welfare (AIHW) (2014) [7] finding that 7.4% of children and adolescents had been assessed as having ADHD in the previous 12 months. 

Anxiety disorders were the next most common (6.9%), followed by major depressive disorder (2.8%) and conduct disorder (2.1%). The forms of emotional disorders in children include depression and anxiety, characterised by the same difficulties which beset adults. Childhood depression and anxiety can manifest itself in school refusal, as a consequence of child maltreatment, or behavioural difficulties. Children with major depression have longstanding psychosocial problems, including family conflict, domestic violence, child abuse, school problems such as bullying and isolation, and may also have parents with mental health problems ([6], p. 88). This may be associated with upheaval in a child’s life, family distress, or external factors causing distress for the child. 

Mental health difficulties for children, if not successfully ameliorated, will continue in their adult lives, albeit often differently expressed. Ongoing conduct disorder is associated with the onset of schizophrenia or sociopathy. Depression and anxiety may remain and be expressed in the range of adult disorders categorised by DSM-5. What is clear is that early intervention into childhood mental health difficulties is imperative, with an integrated service approach, not fragmented and divided as is the current structure throughout much of Australia’s mental health services. 

## 3. Supporting the Child and Family

Clinicians typically intervene at times of transition or crisis; primary healthcare, community health centres, hospitals, networks, mental health services, and welfare or government agencies (such as the child protection service) are places where children may seek help for mental health concerns or where their families may come for help with broader family problems which affect a child’s mental health and wellbeing. 

Working with children in these domains requires specific awareness of how children make sense of mental health problems; what can be expected of them given their age, life, and individual and family circumstances; and what they need from their caregivers for mental health treatment and recovery. The child’s dependency on adults to care for and treat them means that clinicians must work as much with family members as with the individual child, in order to meet the child’s needs and address stresses as well as ensure that the necessary interventions are put in place. Thus, any assessment of needs must be systemic, always being aware that they must be child-centred, as parental needs and perceptions may differ from what is in the child’s best interests. Clinicians in mental health draw not only on theories and knowledge of individual functioning, but also on knowledge of human development, individual and family life stages, and family functioning to effectively and accurately assess the child client’s situation. 

The scope of intervention in child mental health varies according to the nature of the problem experienced, be it crisis intervention, a chronic mental health condition, or a serious illness. A child may be seen where the presenting problem is not mental health-focused (for example, child abuse and neglect, domestic and family violence), but it is important to keep in mind the child’s mental health and assess what impact there is, from the presenting problem, on the child’s mental health and wellbeing. It is this person-in-environment perspective of the mental health experience [8] which characterises clinical practice in this domain. For instance, Reference [9] refers to the need to take a holistic approach to understanding people, their problems, and their reactions. 

Therapeutic interventions when working with children will, as noted, mostly involve working with the family. Moreover, clinicians must be alert to the range of psychosocial issues which affect recovery and actively work to minimise the consequences of illness and improve health outcomes for the child. We are mindful of factors that can impact the capacity to receive care, and the linkages between ‘social illnesses and problems’ and mental illness, such as would be seen in child abuse, exemplifying how psychosocial issues will affect a child and their health outcomes ([10], p. 25).

Children challenged by mental health problems are particularly vulnerable. In working with children and their families, the occurrence of mental health problems gives primacy to child-centred and strengths-based practice, and exemplifies a systems or holistic approach, as the child is centred in a context of relationships which intersect with both the illness experience and their recovery. 

The articles in this special edition canvass a range of issues that intersect with mental illness and children, and offer a variety of experiences by which such illness is understood. The breadth of scholarship herein indicates the many perspectives there are on childhood mental illness, and presents a commendable group of articles to enhance the knowledge and experience of this domain. 
